# Pioneering new paths: the role of generative modelling in neurological disease research

**DOI:** 10.1007/s00424-024-03016-w

**Published:** 2024-10-08

**Authors:** Moritz Seiler, Kerstin Ritter

**Affiliations:** 1 Department of Psychiatry and Psychotherapy, Charité - Universitätsmedizin Berlin, Berlin, Germany; 2 Bernstein Center for Computational Neuroscience Berlin, Berlin, Germany; 3 Hertie Institute for AI in Brain Health, University of Tübingen, Tübingen, Germany

**Keywords:** Generative modelling, Neuroimaging, Neurological disorders, Synthetic data

## Abstract

Recently, deep generative modelling has become an increasingly powerful tool with seminal work in a myriad of disciplines. This powerful modelling approach is supposed to not only have the potential to solve current problems in the medical field but also to enable personalised precision medicine and revolutionise healthcare through applications such as digital twins of patients. Here, the core concepts of generative modelling and popular modelling approaches are first introduced to consider the potential based on methodological concepts for the generation of synthetic data and the ability to learn a representation of observed data. These potentials will be reviewed using current applications in neuroimaging for data synthesis and disease decomposition in Alzheimer’s disease and multiple sclerosis. Finally, challenges for further research and applications will be discussed, including computational and data requirements, model evaluation, and potential privacy risks.

## Introduction

In the last decade, the field of deep learning has led to many breakthroughs in a variety of fields, from computer vision [1–4] to natural language processing [5–7]. Much of this work has been based on a class of models known as discriminative or predictive models (e.g., [1, 3, 8]). However, recently, the class of generative models, in particular deep generative models, gained a lot of interest with seminal work on applications in, e.g., natural language procession (e.g., Generative Pre-trained Transformers (GPTs) [9–12], BERT [13]), chatbots (e.g., ChatGPT [14], Gemini [15]) or text-to-image generators (e.g., DALL-E [16], Stable Diffusion [17, 18]), but also breakthroughs in computational biology like AlphaFold [19] in protein structure prediction or OpenCRISPR in gene editing [20].

What distinguishes these classes of models is the problems they attempt to solve. A discriminative model tries to learn the conditional probability of a target variable given some data. This enables these models to predict the target variable, like a diagnosis or an indicator for disease progression, based on covariates in the data, for instance, a structural magnetic resonance imaging (sMRI) scan. A generative model, on the other hand, attempts to solve a more complex problem. Here, we assume that the data we observe in the real world come from a hypothetical data-generating distribution, which we, unfortunately, do not know. We can only observe samples from this unknown distribution collected in a finite data set. A generative model tries to learn this data-generating distribution, implicitly or explicitly, based on the observed finite data set, which is an ill-posed problem.

Although deep generative models have only recently attracted attention, the field of generative modelling is already decades old, including probabilistic graphical models (PGMs) such as Hidden Markov Models [21], Gaussian Mixture Models [22], or Boltzmann Machines [23]. Deep generative models (DGMs) extend this model class and are a hybrid that combines probabilistic machine learning, probability theory, statistics, and deep learning, enabling complex mapping functions and scalability and, therefore, are interesting for medical applications with high-dimensional data such as neuroimaging data.

In recent years, brain MRI data has become a cornerstone in diagnosing and monitoring neurological diseases associated with measurable brain damage, such as neurodegeneration or inflammatory markers visible in sMRI data [24, 25]. However, the association between these markers and their exploratory value, e.g., for disease severity or progression, might be limited [26], requiring an understanding of the disease structure and its interconnected factors. Given the success in a plethora of applications for different modalities and the need for a deeper understanding of disease drivers, the question arises as to the potential and capabilities of these generative models when applied to neuroimaging data. The potential of generative models by learning the data-generating distribution might enable these approaches to tackle current problems, such as high costs or high privacy protection requirements, in the collection of medical data. At the same time, learning the data-generating distribution implies learning the individual factors in a patient or a specific neurological disorder and the structure underlying the data-generating distribution. In addition, a multimodal extension that combines the different systems within a patient could enable us to create a complete digital twin of a patient, a digital replication, in the future. The concept of the digital twin originates from industry or manufacturing practice, where sensor data is used to build a digital replica of a system, linking the physical system to a virtual system [27]. In clinical decision-making, this could help to run simulations like disease progression or the individual effects of interventions, such as a change in treatment or medication, in a patient without actually performing them in real life. These potentials of digital twins are viewed as a future approach to precision medicine; for a review, see Sun et al. [28], Katsoulakis et al. [29]. A specific example of a model-based digital twin is the virtual brain [30, 31] - a generative brain network - enabling brain function exploration and hypothesis testing. These brain networks integrate structural connectivity information into a probabilistic framework that allows testing diagnostic and therapeutic interventions (see, e.g., [31] for a potential clinical decision-making application in estimating the epileptogenic zone).

This review provides an overview of the current state of the art in generative modelling applications in neuroimaging-based neurological disease research and their future potential. In the “Generative modelling” section, we will introduce generative models with the key concept, popular models, and methodological promises for use in neuroimaging, namely data generation and representation learning. In the “Clinical applications of generative modelling” section, we will review current applications of generative models in synthetic data generation and disease decomposition in Alzheimer’s disease (AD) and Multiple Sclerosis (MS). Finally, challenges and implications of current applications of generative models in neuroimaging are discussed in the “Challenges” section.

## Generative modelling

To make applications and promises of generative modelling more accessible, we briefly introduce some basic concepts of this model class. Generative models describe a class of different modelling approaches, which can be divided into the more classical PGMs and the recent DGMs, which use neural networks for mapping, although hybrids are possible. Within this review, we focus on the popular recent DGMs. In this section, we will give a high-level, intuitive introduction to some popular generative modelling approaches and goals in terms of application in neurological disease research using neuroimaging data; for a more detailed and technical introduction, see, e.g., [32, 33].

### Overview

In mathematical terms, a generative model is a probability distribution $p(\textbf{x})$ or $p(\textbf{x},y)$ with data $\textbf{x} \in \mathcal {X}$ and labels $y \in \mathcal {Y}$. To better understand the problem definition in generative modelling, we assume that the data we observe in the real world comes from a data-generating distribution, which is unknown. We are only able to observe samples or realisations of this unknown data-generating distribution, which we collect in finite datasets. The goal of generative modelling is to learn this unknown probability distribution, i.e., a generative model is used to understand how the data was generated in the first place. A discriminative model or predictive model, on the other hand, is mathematically a conditional distribution $p(y|\textbf{x})$, i.e., we use a discriminative model to learn a relationship or mathematical function between some data $\textbf{x} \in \mathcal {X}$ and labels $y \in \mathcal {Y}$ in a regression or classification. This class of models can be considered a special case of a generative model, as we will see in the following example.

**Fig. 1:**
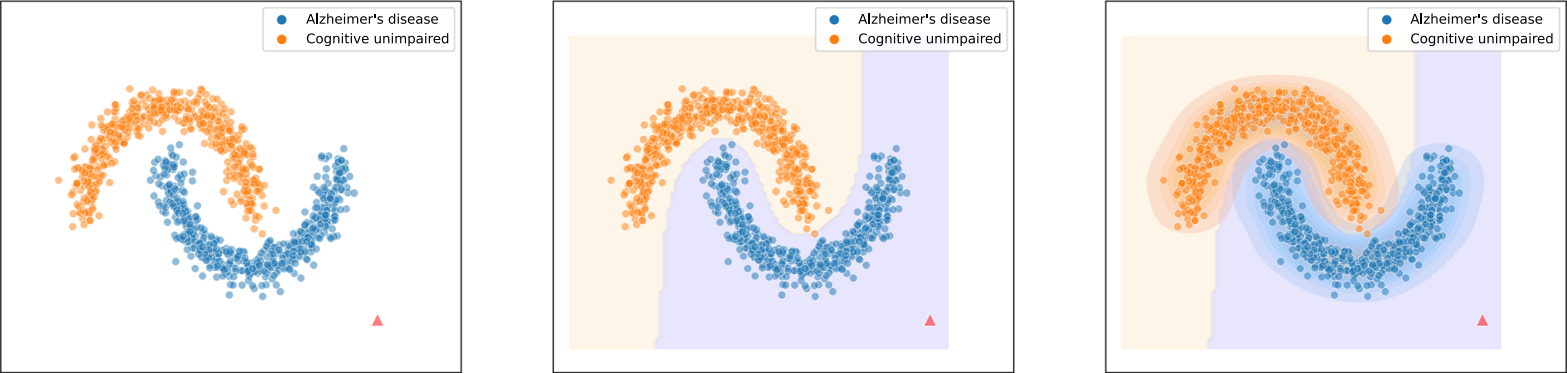
Example data of patients with AD (blue) and cognitive unimpaired subjects (orange) with an unlabeled new observation *(red triangle)(left)* and a discriminative *(middle)* and generative approach *(right)* to medical decision-making

Imagine we have a dataset which contains both healthy subjects *(orange)* and subjects diagnosed with AD *(blue)* (see Fig. 1). Both groups are similar within the group but different between the groups, as can be seen from the non-overlapping clusters. A new observation *(red triangle)* is added to the dataset that does not lie in one of the clusters. Now, we train a discriminative model *(middle)* and a generative model *(right)* for an informed decision.

After training the discriminative model $p(y|\textbf{x})$, we receive a decision boundary which separates the two clusters. As can be seen, the new observation is on the blue side and far from the decision boundary, i.e., the model assigns this new observation a higher probability of being diagnosed with Alzheimer’s disease and is certain about this decision, $p(y=\textit{AD})|\textbf{x}=\textit{red triangle})$ is high. The generative model *(right)* considers the joint distribution $p(\textbf{x},y)$ which can further be decomposed into $p(\textbf{x},y) = p(y|\textbf{x})p(\textbf{x})$. In addition to the decision boundary $p(y|\textbf{x})$, we fit a distribution $p(\textbf{x})$. As we have seen before, the new observation is on the blue side and far from the decision boundary, i.e., $p(y=\textit{AD})|\textbf{x}=\textit{red triangle})$ is high; however, it is also far from the clusters and, therefore, far from the training data, i.e., $p(\textbf{x}=\textit{red triangle})$ is low. This results in a low joint distribution $p(\textbf{x}=\textit{red triangle},y=\textit{AD})=p(y=\textit{AD})|\textbf{x}=\textit{red triangle})p(\textbf{x}=\textit{red triangle})$ indicating an uncertain decision. Learning the data distribution $p(\textbf{x})$ gives information on the structure of the data.

This indicates the limitation of discriminative models in medical decision-making. It lacks understanding of the environment and cannot express uncertainty directly, which is essential in medical decision-making. This comparison with discriminative modelling already shows that the goal of generative modelling is much more ambitious.

### Model types

The first type of DGM is the group of *autoregressive models* (ARMs). This group of models computes the data distribution $p(\textbf{x})$ over *T* variables in an autoregressive form as

1 $$\begin{aligned} p(\textbf{x}) = p(x_0) \prod _{t=1}^T p(x_t|\textbf{x}_{0:t-1}) \end{aligned}$$

where $\textbf{x}_{0:t-1}$ is all the data $\textbf{x}$ up to *t* using the chain rule of probability. Since each conditional distribution becomes more complex with increasing *t*, assumptions on these distributions like Markovian assumption $p(x_t|\textbf{x}_{0:t-1})=p(x_t|\textbf{x}_{t-1})$ or latent variables $z_t \in \mathcal {Z}$, which compresses information about the past (e.g., recurrent neural networks (RNNs) or long-term shot memory neural networks (LSTMs) [34]), are introduced to address tractability issues of the general form in Eq. 1. Alternatively, neural networks are used to implicitly learn a mapping between the past and future elements within $p(x_t|\textbf{x}_{1:t-1})$ like the neural autoregressive density estimation (NADE) [35], causal convolutional neural networks [7, 36], or transformer models [6]. This autoregressive form makes them suitable for sequence modelling, e.g., language [11], audio [7], etc., where images can also be seen as a sequence of pixels or voxels with a spatial dependency structure [36]. While ARMs allow exact likelihood estimation, the sequential nature makes sampling from these models slow.

In contrast, the *variational autoencoder* (VAE) [37] is a deep latent variable model assuming that the observed data $\textbf{x}$ is generated by a non-observable latent random variable $\textbf{z} \in \mathcal {Z}$, which usually lies in a lower dimensional latent space $\mathcal {Z}$. These can be seen as hidden factors which are essential to the generation of the observed data. The general definition is

2 $$\begin{aligned} p(\textbf{x}) = \int _z p(z)p(\textbf{x}|z) dz \end{aligned}$$

with *p*(*z*) being a prior distribution on the latent variable *z* and $p(\textbf{x}|z)$ the likelihood of the data $\textbf{x}$ given the latent code *z*. This would allow us to obtain the posterior distribution $p(\textbf{z}|\textbf{x})$ for inference, but computing $p(\textbf{x})$ is in general intractable and cannot be evaluated. To address this problem, the VAE uses a probabilistic encoder or recognition model $q(\textbf{z}|\textbf{x})$, which is parameterised by a neural network, and a decoder or the generative model $p(\textbf{x}|\textbf{z})$, which is parameterised by a neural network, too. Here, $q(\textbf{z}|\textbf{x})$ is a variational posterior distribution, typically a multivariate Gaussian with a diagonal covariance matrix, used to approximate the intractable posterior $p(\textbf{z}|\textbf{x})$ [37]. The input $\textbf{x}$, like an image, is used by the probabilistic encoder to learn a distribution over the latent factors $q(\textbf{z}|\textbf{x})$. Based on samples $\textbf{z}$ from the variational posterior distribution, the decoder $p(\textbf{x}|\textbf{z})$ is used to reconstruct the original input $\textbf{x}$. Although the VAE is a popular approach for representation learning (see the “Representation learning” section), it only provides a lower bound to the likelihood $p(\textbf{x})$ and does not allow for high-resolution reconstructions; so, generated images appear blurred. Extension of this method focus, e.g., on disentangled latent variables [38], sparsity in the latent factors [39], or hierarchical structures for high-quality data generation [40].

*Generative adversarial networks* (GANs) [2] are a game-theoretic-based approach to generative modelling in which a generator network and a discriminatory network compete in a game. The generator network generates samples $x=g(\textbf{z})$ from noise $\textbf{z}$, while the adversarial discriminator network distinguishes between samples from the dataset and samples generated by the generator network. By training both networks simultaneously, the generated data from the generator network becomes indistinguishable from the real data. In contrast to ARMs, VAEs, or flow-based models, the GAN is an implicit generative model, meaning that the likelihood is not modelled directly [41]. The most popular application of this type of generative model is image data [42–44] because it was the first successful approach enabling synthesising realistic images. But also further applications to video [45], audio [46], and text [47], although the discrete nature of text data makes applications of GANs challenging, were introduced. Despite their impressive synthesising abilities, training this type of deep generative model is challenging due to unstable training behaviour [48] and mode collapse [49].

Another type of deep generative model to construct flexible, learnable probability distributions is *normalising flows* [50], based on the change-of-variable formula. They provide a principled way to describe the data distribution $p(\textbf{x})$ by an invertible transformation $\textbf{x}=f(\textbf{z})$, such that $g(\textbf{x})=f^{-1}(\textbf{x})=\textbf{z}$, of a known, simple source of noise $\textbf{z}$, e.g., a standard normal distribution $\textbf{z} \sim \mathcal {N}\left( 0,I\right)$. The change-of-variable formula is defined as

3 $$\begin{aligned} p(\textbf{x})=p(g(\textbf{x}))\left| \det \textbf{J}_{g(\textbf{x})}\right| = p(\textbf{z}) \left| \det \textbf{J}_{f(\textbf{z})}\right| ^{-1} \end{aligned}$$

where $\textbf{J}_{f(\textbf{z})}$ is the Jacobian matrix of *f* evaluated at $\textbf{z}$. This means when we want to sample from $p(\textbf{x})$, we first sample $\textbf{z}$ from a simple known distribution, e.g., $\textbf{z} \sim \mathcal {N}\left( 0,I\right)$, and transform it using $\textbf{x}=f(\textbf{z})$. To compute the distribution $p(\textbf{x})$, we can then use the fact that $f(\cdot )$ is invertible to normalise the data distribution by mapping it back to the simple distribution $p(\textbf{z})$. In these normalising flow models, the invertible functions $f(\cdot )$ are modelled using neural networks. Given a flexible enough mapping $f(\cdot )$, normalising flows can approximate any smooth distribution [50]. Popular applications for normalising flows are density estimation like non-linear independent components estimation (NICE) [51] or real-valued non-volume preserving (real NVP) and data generation of images [52, 53], video [54], audio [55], or text [56]. Despite their flexibility and ability to allow exact likelihood estimation, normalising flows do not compress the data like a VAE by default and thus require a lot of computation.

**Fig. 2:**
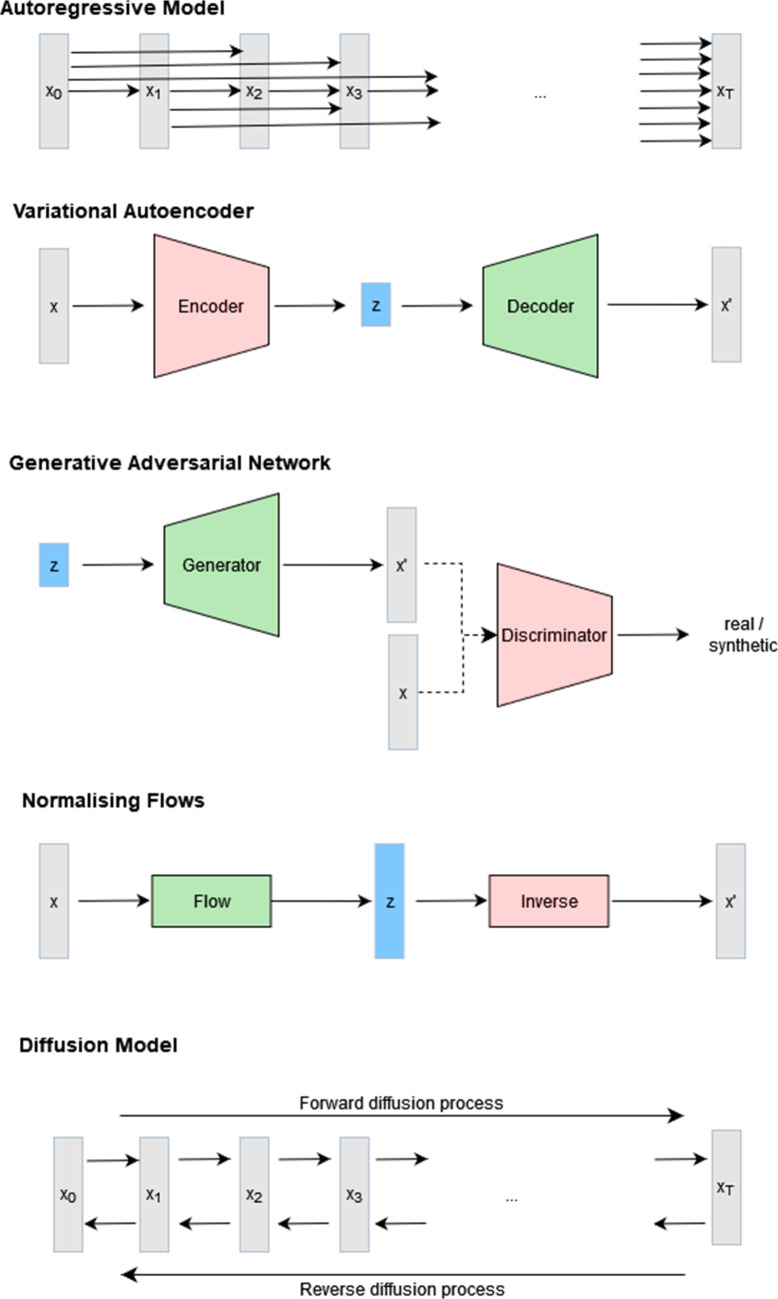
Schematics of generative models: ARM, VAE, GAN, normalising flows, and diffusion model. Here, *x* denotes the observed data, $x'$ the generated data sampled from the model, and *z* the latent factor

*Deep diffusion models* [57–59], inspired by non-equilibrium statistical physics, iteratively destroy the structure of the data through a forward diffusion process (by adding noise) to learn a reverse diffusion process to restore the original data structure. In this forward diffusion process, the observed data $x_0$ is passed through a stochastic encoder $q(x_t|x_{t-1})$, creating a noisier version of its input until after *T* steps, the resulting output $x_T$ follows a reference distribution, e.g., $X_T \sim \mathcal {N}\left( 0,I \right)$. In the reverse process, the noisy encoder output $x_T$ is passed through a decoder $p(x_{t-1}|x)$, which learns to remove the noise stepwise until, after *T* steps, the original input $x_0$ is generated. While these methods are primarily used to generate high-resolution images (e.g., [16, 17]), applications to text were also introduced (e.g., [60]). Unlike VAEs or GANs, diffusion models are less effective for representation learning since they do not necessarily provide a good, compressed latent representation. Popular diffusion models are, for example, denoising diffusion probabilistic models (DDPMs) [57], the closely related denoising diffusion implicit models (DDIMs) [61], or latent diffusion model (LDMs) [17]. Schematics of the described models are shown in Fig. 2.

All these generative approaches are based on statistical associations, which, due to potential spurious associations, harbour the risk of learning possible biases without any causal significance. Thus, recent research focuses on introducing causality into generative modelling to improve the interpretability and robustness of these models (e.g., [62]). In particular, the structural causal model (SCM) formalism [63] describes the data-generating process with a set of variables through a causal mechanism. This allows causal generative models to predict not only the effect of an intervention but also reasoning about counterfactuals.

### Goals of generative modelling

**Fig. 3:**
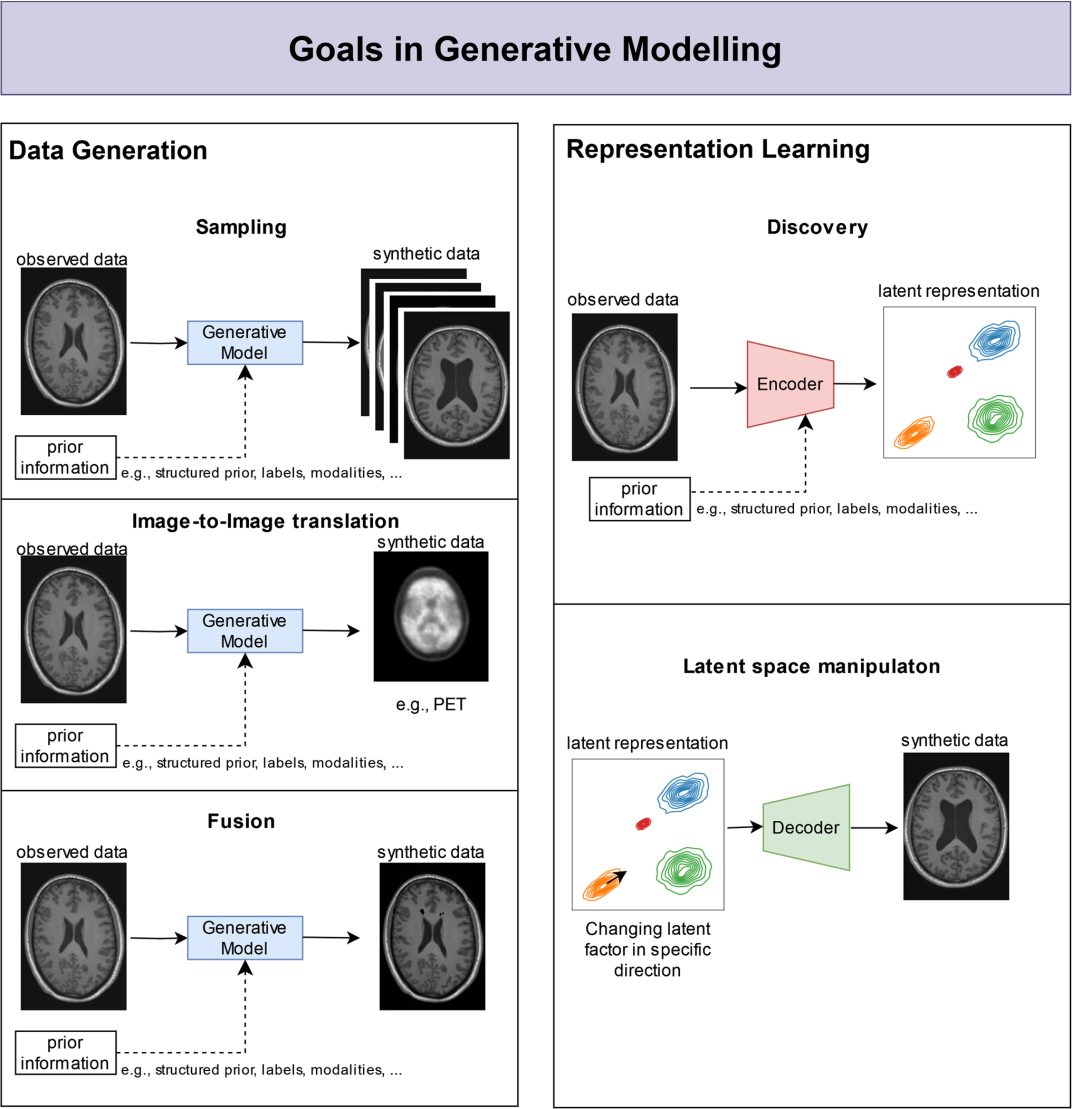
Potential applications in data generation and representation learning in neurological disease research. For specific examples, see the “Clinical applications of generative modelling” section

Motivated by the ability of generative models to implicitly or explicitly learn the data-generating distribution, we selected two applications that have potential in medical imaging in the context of neurological diseases. This section introduces the concepts of these applications, namely, the data generation and the ability of some generative models to learn a representation of the observed data. Potential applications are visualised in Fig. 3.

#### Data generation

One of the main goals of generative modelling is to generate new data. Once the data distribution *p*(*x*) is learnt, we can draw new samples from it. To control the generated output, conditional generative models $p(\textbf{x}|\textbf{c})$ can be used, where $\textbf{c}$ is the condition across modalities like text-to-image, image-to-text, image-to-image translation, or text generation. When data is generated to further train other models, this created data are called synthetic data [64], which is currently considered a future enabling technology. The generated synthetic data must have the same statistical properties as the observed real-world data. This offers the potential for a tremendous impact on challenges like privacy, fairness, and small sample sizes [64]. For example, sensitive information, like patient records, cannot easily be shared due to regulations like the General Data Protection Regulation (GDPR) or the Health Insurance Portability and Accountability Act (HIPAA). The potential of synthetic data, which does not correspond to a real patient, allows bypassing these legal frameworks and facilitates cross-border collaboration in research. Moreover, the costs associated with data collection could be extremely high and prevent the collection of large data sets. Here, generative models might offer a scalable solution to generate large amounts of data. The desired behaviour of a generative model for the goal of data generation is the ability to generalise, which, depending on the data distribution, means that the generative model might have to be trained on a large dataset before. For natural images, e.g., Kadkhodaie et al. [65] showed that diffusion models require large datasets to transition to a strong form of generalisation instead of pure memorisation of the training data. Thereby, the size of the dataset depends on the image size and the data complexity relative to the model capacity [65, 66]. Although generative models and synthetic data have the potential for transformation, the statistical properties and the effects of synthetic data on further model training still require further research (see, e.g., [67–70]).

The potential of data synthesis in generative models is not limited to the creation of large data sets but also to a possible cost-effective extension of the observations by synthesising additional modalities (cross-modality translation or fusion). Furthermore, the generative aspect is also crucial for the replication of patients in digital twins and the simulation of possible effects on their systems. Simulating the development of a digital twin enables the simulation of effects such as treatment effects, disease progression or potential outcomes, enabling personalised medical decision-making. [29].

#### Representation learning

The motivation for representation learning in generative modelling lies in the assumption that the generation of data implicitly requires an understanding of the data and its generative process. While fully-observed models, like ARMs, can learn useful representations directly without relying on latent factors [71, 72], latent factor models, like VAEs or GANs, are a popular approach to learning a representation of the observed data. These models use the idea of latent variables $\textbf{z} \in \mathcal {Z}$, which generate the observable data $\textbf{x}$, i.e., $p(\textbf{x}|\textbf{z})$. In these cases, Bayes’ rule can be used to find a representation by considering the posterior distribution on the latent variable given the data $p(\textbf{z}|\textbf{x}) \propto p(\textbf{z}) p(\textbf{x}|\textbf{z})$ with these latent factors usually as low-dimensional patterns. These patterns can, for instance, be semantic attributes like information on hair colour, hair style, eye colour, facial expression, etc., in a person’s portrait. However, the interpretability of these generating latent factors is not necessarily guaranteed. This sometimes leads to the trade-off between imposing structure on the generative process, like sparsity [39] or disentangled factors [38, 73], versus learning the structure only from the observed data [37]. When learning disease-specific factors, the individual generating factors should be independent and interpretable if possible. In this regard, [74] have shown that learning independent factors is impossible without an inductive bias or supervision.

In contrast to these DGMs, which rely on statistical associations, representation learning can be viewed from a causal perspective to learn the true causal mechanisms of the data-generating process [75]. This causal approach can address challenges like identifiability, robustness, or biases that non-causal representation learning suffers from [76, 77]. While causal discovery methods attempt to identify the causal structure of the data-generating distribution, these approaches are not scalable and are of limited use in neuroimaging [76]. However, SCMs that require knowledge of the causal relationships can use the ability to learn representations of these DGMs to model causal effects. Based on these SCMs, we can predict the effect of interventions and determine hypothetical causes of effects through counterfactuals (*what if?*) [78]. These capabilities of SCMs are essential for designing a digital twin and informed medical decision-making.

On the one hand, learning representations in generative models can help to discover the underlying structures of the data-generating distribution, e.g., individual factors of a disease and their structure. On the other hand, effects can be measured, in particular, when embedded in a causal framework by manipulating learnt representations to identify possible causes of these effects.

## Clinical applications of generative modelling

Although the field of generative models is very young, several applications based on generative models in neuroimaging have been introduced (for reviews, see [79–82]). However, specific applications for neurological diseases - potentially due to small sample sizes - are still rather rare; nevertheless, we will review these concerning synthetic data generation (“Data generation” section) and disease decomposition (“Representation learning” section) as visualised in Fig. 3 for applications in Alzheimer’s disease (AD) and multiple sclerosis (MS).

### Synthetic data generation

As described in the “Data generation” section, the generation of data is one of the primary goals of generative models, making them particularly interesting for medical applications due to, e.g., costs and time consumption associated with data collection, sensitivity of patient data and privacy protection requirements, or simulations within a digital twin. A generative model that has learnt the data-generating process might be able to address these issues by generating synthetic data, i.e., statistically indistinguishable from the observed real-world data, at scale. In disease-specific applications of the generative models, it is crucial not only to preserve the healthy but also the pathological morphology. Within synthetic data generation, we focus on applications in synthesis by sampling from the learnt data-generating distribution using a single modality, image-to-image translation, and data fusion of pathological data.

Applications for disease-specific synthetic data generation by sampling from the learnt data-generating distribution for a specific modality can be divided into generating longitudinal or cross-sectional synthetic data. In general, most recent applications in this regard are in the field of Alzheimer’s Disease (AD) [83–89], a neurological disorder that is the main cause of dementia in the elderly with rather clear neurobiological correlates (i.e., neurodegeneration and resulting atrophy starting in the hippocampus). One reason for this might be the existence of the Alzheimer’s Disease Neuroimaging Initiative (ADNI) database [90], a, from a medical perspective, relatively large open database, which, in general, considerably boosted the number of machine learning studies.

In neurological disease research, longitudinal data is of uppermost importance since it can provide spatiotemporal information on the disease process and its dynamics. However, collecting large longitudinal datasets is costly and time-consuming, with inherent risks, such as dropouts. Spatiotemporal generative models have been proposed to address these issues. Nevertheless, the generation of longitudinal synthetic neuroimaging data is computationally expensive due to the high dimensionality and the additional time dimension. Therefore, 2D slice-based methods were introduced by Jung et al. [83] and Ravi et al. [89], which merge those individual slices into a 3D MRI or 4D MRI (3D MRI + time) to reduce the computational requirements. To prevent potential artefacts between these 2D slices, these GAN-based methods use specific modules for spatial alignment in the network architecture. Although these approaches generate high-quality synthetic data with plausible pathomorphological changes, for instance, the 4D-DANI-Net shows differences in capturing neurodegeneration depending on the size of specific brain areas [89], which might be a consequence of the slice-based approach. In contrast to these approaches, Puglisi et al. [91] proposed Brain Latent Progression (BrLP), an LDM-based model, for consistent spatiotemporal synthetic data generation as they enable high-fidelity data generation. In addition, BrLP is capable of including subject-specific (age, sex, and cognitive status) and progression-related (volumes of the hippocampus, cerebral cortex, amygdala, cerebral white matter, and lateral ventricle) as prior information. To leverage prior knowledge of a disease, disease progression models can be used to model more accurate trajectories aligned with the patient’s disease history based on these disease-related variables. For training, 11730 T1w 3D MRI scans from 2850 subjects (ADNI-1, ADNI-2, ADNI-3, ADNI-GO, OASIS-3, and AIBL [92]) were used. The evaluation of this approach included tracking changes in the AD-related brain regions, which could be predicted more accurately and consistently by BrLP compared to sequence-aware diffusion model (SADM) [93], DANI-Net [89], and CounterSynth [94], indicating the importance of longitudinal models and informed prior information. Nevertheless, problems were identified in data synthesis at the tails of the distribution of conditional variables, i.e., underrepresented data points in the training data set. Similarly, Zhao et al. [95] use a 3D patch-based multi-information generative adversarial network (mi-GAN) in combination with a 3D DenseNet to model disease progression and diagnosis classification in patients with AD. Progression was defined as morphological changes four years after the baseline visit using data synthesis based on the mi-GAN framework. In addition to the T1w MRI data, information on age, gender, education level, and the APOE $\varepsilon 4$ allele status at the baseline visit was used as input. The two networks were trained and evaluated separately, without diagnosis-guided generation, on subjects from the ADNI-GO and ADNI-2 datasets (813 subjects in total) and tested independently on 48 subjects from the OASIS dataset. The results showed a better generation capability compared to the cGAN framework proposed by Yan et al. [88] and comparable prediction performance of future diagnoses based on the generated images compared to the real T1w MRI data.

Although longitudinal data is of enormous relevance for neurological disease research, models for the generation of cross-sectional data offer the possibility to generate data without explicitly modelling the temporal dimension and its complexity, resulting in simpler models for synthetic data generation. Instead of GAN-based generative models, ARMs [84] and, in particular, diffusion models [86] are used to generate high-quality MRI data. Tudosiu et al. [84] introduced an ARM combining a vector-quantised VAE (VQ-VAE) and a transformer model to generate morphology-preserving synthetic data for healthy subjects and patients with AD. For this, the VQ-VAE was first pre-trained on 31740 T1w MRI scans of healthy subjects from UK Biobank [96] and then fine-tuned on the pathological data ($n=648$) from the ADNI database. In the second step, a transformer model was trained on the latent representation of the VQ-VAE of different subgroups to evaluate the morphological differences of the synthetic data. For evaluation of the synthetic MRI, Voxel-Based Morphometry [97] was performed and showed an overall similarity between the real-world observations and the generated synthetic data, although significant differences in cerebral spinal fluid (CSF) and grey matter (GM) volumes were observed. The diffusion model-based approach, introduced by Peng et al. [86], is based on a 2D conditional diffusion probabilistic model (cDPM) that can learn the spatial dependencies between slices to generate high-quality synthetic 3D MRI. A qualitative analysis of the generated images showed that this method, compared to similar GAN-based and diffusion-based methods, was capable of synthesising data similar to that in the training data without any structural differences, indicating the imminent importance of further research in the statistical properties of synthetic data.

In addition to generating synthetic data sampled from the learnt data-generating distribution, image-to-image translation can be used to synthesise costly and hard-to-access data, such as synthesising expensive positron emission tomography (PET) from relatively inexpensive and available MRI data [87, 88]. This synthetic PET might be beneficial for diagnosing and monitoring the progression of a disease [98]. For this image-to-image translation, Yan et al. [88] proposed a cGAN for a T1w-to-$^{18}F-$florbetapor PET scan translation. The used dataset was relatively small, containing 79 subjects with either a developmental mild cognitive impairment or a stable mild cognitive impairment diagnosis with pairs of PET and T1w MRI scans. In contrast, Lin et al. [87] introduced a 3D Reversible Generative Adversarial Network (RevGAN) for this application, which was trained on a larger database containing 2076 images from 1086 subjects in the ADNI-1, ADNI-2, ADNI-3, and ADNI GO databases. The synthesised PET scans were then evaluated on multimodal classification tasks (cognitively unimpaired vs. dementia or developmental mild cognitive impairment vs. stable mild cognitive impairment) with different combinations of MRI, PET, and synthesised PET, showing on-par performance compared to models trained on real-world observations. Pan et al. [99] merge this two-step approach of data generation and diagnoses in a single disease-image specific deep learning (DSDL) framework, which combines a Disease-image-Specific Network (DSNet) to model the disease-image specificity and a Feature-consistency Generative Adversarial Network (FGAN) for data synthesis and imputation of missing neuroimaging data focusing on disease-relevant brain regions. This framework was trained and evaluated on the ADNI and AIBL databases, showing on-par results in data synthesis and state-of-the-art performance in diagnosis classification (cognitively unimpaired vs. dementia and progressive mild cognitive impairment vs. stable mild cognitive impairment). Likewise, Gao et al. [100] introduced a task-induced pyramid and attention generative adversarial network (TPA-GAN) to preserve relevant image features for imputation of PET images based on T1w MRI, which extends the GAN framework with an additional path-wise transfer dense convolution network (PT-DCN) discriminator for multimodal diagnosis classification to guide the data synthesis. The joint framework was trained on the ADNI-1 and evaluated on the ADNI-2 database. Again, the results show comparable performance in the imputation performance of the approach and similar or improved performance in the classification performance of the diagnostic labels, with the improvement in classification performance due to the multimodal approach being evident. In multiple sclerosis (MS), PET has become a valuable tool for measuring in-vivo changes in myelin content [101]. Therefore, the synthesis of PET from multi-sequence MRI is of paramount importance for understanding the underlying mechanisms of MS pathology. Wei et al. [102] proposed a conditional flexible self-attention GAN (CF-SAGAN) to predict a parametric map of $\left[ ^{11}\text {C}\right]$PIB PET to derive the longitudinal myelin content changes from multisequence Diffusion Tensor Imaging (DTI). An evaluation of the predictions showed increased performances for static and dynamic demyelination/remyelination. Nevertheless, due to the selected attention regularisation in this approach, a trade-off between global and local image quality is required.

In contrast to applications in AD, applications in MS focus, in particular, on lesions, inflammatory markers which are a hallmark of the disease [103–106]. These are GAN-based approaches to learn the semantics of pathological subjects and synthesise the lesion patterns on healthy subjects by fusing the data. This fused data can then be further used for data augmentation [103, 104] or the creation of counterfactuals to understand the disease process in MS [107]. The datasets for these applications were relatively small, typically $n\le 135$, due to a lack of large open databases in MS.

**Fig. 4:**
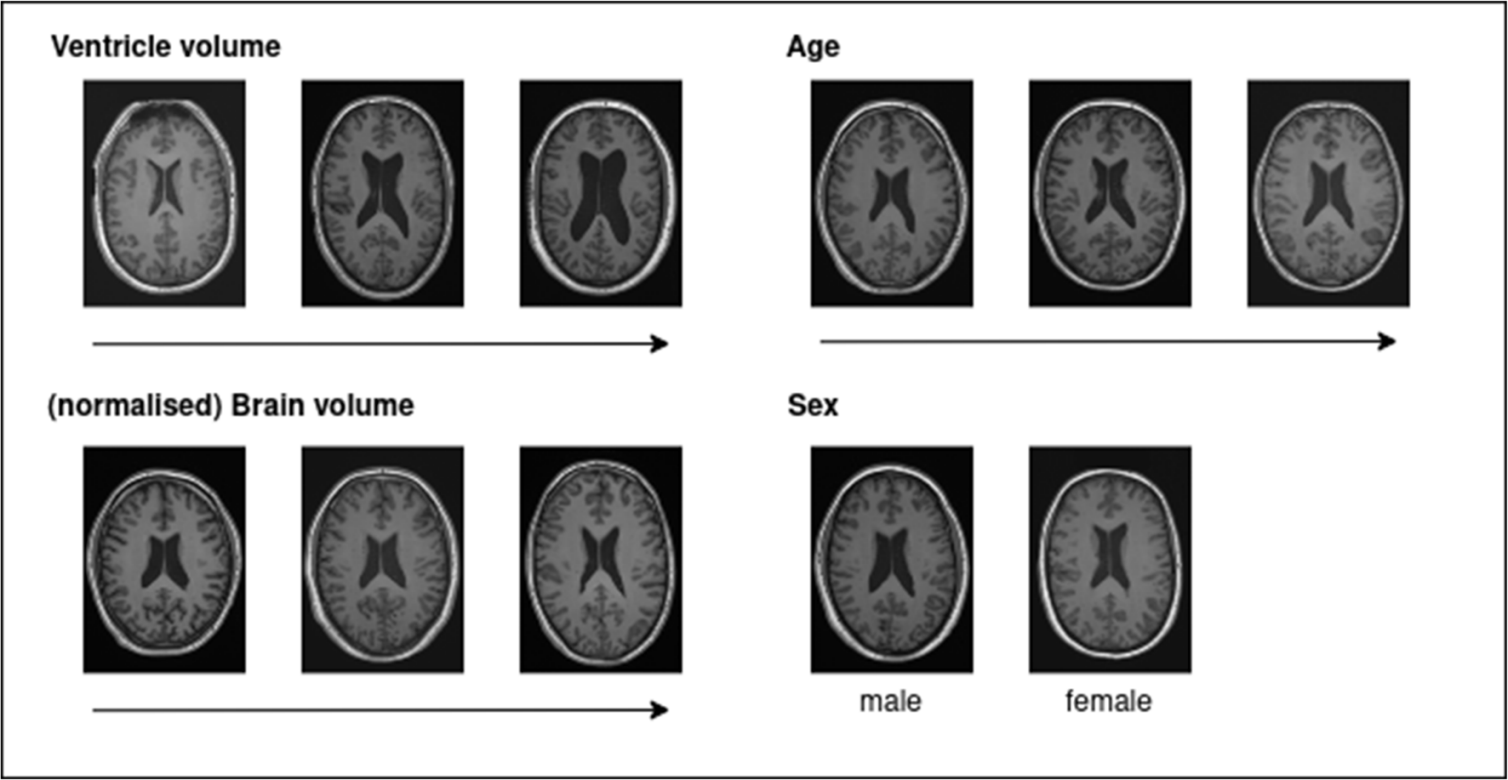
Synthetic images generated by conditional sampling with varying ventricle volume *(top left)*, (normalised) brain volume *(bottom left)*, age *(top right)*, and sex *(bottom right)* from the LDM proposed by [108] while keeping the other variables constant

Although not a direct application to pathological data, the recent introduction of 3D diffusion models has led to the synthesis of impressive, high-resolution 3D MRI data. Pinaya et al. [108] introduced a latent diffusion model capable of generating high-resolution 3D T1w MRI scans. For this data generation, the generative model was trained on 31740 participants of the UK Biobank, being able to generate a 3D T1w MRI conditioned of the sex, age, brain volume, and ventricle volume of a data subject. Variations of the conditioning variables brain volume, ventricle volume, age, and sex are shown in Fig. 4.

Based on this generative method, a dataset of 100000 synthetic 3D T1w MRI scans was made publicly available [108]. Although the model generates high-fidelity data (1*mm* isotropic) with realistic variations and the ability to extrapolate the conditional variables, this diffusion model is not able to consider a temporal dimension, i.e., it is not able to generate longitudinal data, which is of importance for disease progression or monitoring. While an application to pathological data would be conceivable, the lack of large datasets is probably the bottleneck for such an application. An extension to this approach, Fernandez et al. [109] proposed brainSPADE3D, a generative model which can generate labels and 3D brain MRI combined with segmentation, allowing for conditioning on pathological phenotypes (tumour, oedema, white matter hypointensities (WMH), gadolinium-enhancing (GDE), non-enhancing (nGDE)) and contrasts (T1w, T2w, Fluid-Attenuated Inversion Recovery (FLAIR)). The training data for this generative model consists of 630 subjects from SABRE [110], 66 subjects from ADNI-2 [90], and 103 subjects from the BRATS dataset [111]. Although this training set is relatively small, the generative model seemed to have learnt disentanglement of the pathologies, boosting, for instance, the performance for WMH segmentation in the appearance of tumours. However, in contrast to Pinaya et al. [108], the resolution of the generated MRI data is lower (2*mm* isotropic) due to computational limitations, the extrapolation capability of the generative model is limited, and the non-consideration of explanatory variables, e.g., sex or age, in the conditioning set leads to lower variability in the generation.

Overall, all these applications demonstrate the ability of generative models to produce plausible synthetic data up to high-quality MRI, which could solve current problems, such as data scarcity in particular. However, this requires further investigation of their statistical properties beyond variability, comparison with the original data, or qualitative optical comparisons [86]. While most applications use a GAN-based model, which can be unstable in training [2], the recently introduced diffusion-based approaches show impressive image quality, however, coming at high computational costs [108]. Here, current slice-based methods such as [86], which enable the synthesis of high-fidelity 3D images, are promising options that trade off image quality, computing costs, and training stability. For future applications, such as digital twins, image-to-image translation can help generate a more comprehensive image of the patient for simulation to understand the underlying mechanisms of a disease. Furthermore, these applications show the importance of structured prior information about the disease or confounding factors to learn plausible effects.

### Disease decomposition

The ability of some generative models, namely VAEs and GANs, to learn a latent representation of the data might enable us to identify underlying associations and generative factors in pathological data to understand the underlying mechanisms of disease. While disentanglement is often used in harmonisation (e.g., multicenter [112] or multi-sequence [113]), it can also be used to decompose generative neuroanatomical factors of disease to derive distinct disease subtypes and discover brain endophenotypes. Heterogeneity in the presentation of neurological disorders and overlapping factors, such as ageing [114] or confounding factors, pose challenges to plausible interpretability and clinical meaningfulness in the identification or discovery. Disentangling this heterogeneity and overlapping factors might enable the identification of interpretable factors that generate pathological processes. This latent structure can then be further manipulated by altering factors in a particular direction to determine the effects of such a specific change.

Such applications of discovery to AD [115–119] attempt to model the course of the disease to characterise the heterogeneity in neurodegeneration and to separate effects, such as the ageing effect. Yang et al. [115] used a semi-supervised clustering-generative adversarial network (Smile-GAN) in a weakly-supervised approach to identify disease subtypes with distinct neuroimaging signatures. Training the model on derived volumetric data of atlas-based regions of interest (ROI) from 8146 T1w MRI from 2832 subjects across two harmonised datasets (ADNI, Baltimore Longitudinal Study of Aging [120]) including only data from cognitive unimpaired and patients with AD enables disentangling pathologic neuroanatomical heterogeneity. Smile-GAN identifies four distinct patterns of neurodegeneration from mild to advanced atrophy that span the entire spectrum of patients with AD, leading to two different trajectories of disease progression. These identified subtypes showed statistical associations with cognitive test performances, executive dysfunction, or memory impairment. An extension to this was introduced with semi-supervised representation learning via GAN (Surreal-GAN), using a continuous process for disease heterogeneity [121]. This approach discovered factors of diffuse cortical atrophy and focal atrophy in medial temporal lobes in patients with AD, showing differences in their correlations to, e.g., lesion volumes, presence of hypertension, CSF-Tau, or APOE-E4 alleles. A further multimodal extension of this is the gene-guided weakly-supervised clustering via generative adversarial networks (Gene-SGAN) [117]. Yang et al. [117] proposed a generative model that extends the previous approaches, using only volumetric brain information, with genetic data. The model was trained on T1w-derived volumetric data for 144 ROI and 178 AD-associated single nucleotide polymorphisms (SNPs) as genetic features of 28858 subjects from ADNI and the UK Biobank. Gene-SGAN identified four AD-related subtypes with distinct atrophy signatures and associations with known AD-related genetic variants. Additionally, the model identified five clinically distinct hypertension-related subtypes indicating associations with blood pressure, other comorbidities, and neuroanatomical changes. Overall, the multimodal approach enabled the identification of subtypes, which could not be identified on image data alone based on Smile-GAN. However, these approaches are based on image-derived volumetric data, which highly compress the overall image information. As a result, this leads to a lower complexity of the models, which is justifiable due to the amount of data. Nonetheless, it ignores more granular potential local effects, which cannot be expressed in volumetric ROI data.

In contrast to these ROI-based discovery approaches, 2D slice-based methods for discovery were introduced. For instance, Couronné et al. [116] proposed a VAE-based DeepSet network to disentangle inter-patient variability to learn disease stages in AD. Different from [115, 117, 121], this longitudinal generative model was trained on 2D T1w MRI data of 356 MCI converters with 1898 imaging visits from the ADNI database. This DeepSet approach identified two latent factors showing linear associations between the disease stage and disease severity proxies, like the ratio of ventricle and brain volumes and clinical parameters, e.g., APOE4 (1 or 2 alleles), in a post-hoc correlation analysis. Using a hierarchical VAE, Hu et al. [118] propose an approach to disentangle fine-grained disease pathology from subject-specific anatomy in sMRI based on structured priors. The generative model was applied to both central 2D slices of T1w MRI of 864 subjects from ADNI and central 2D slices of FLAIR of 815 subjects from a proprietary MS dataset. In both sequences, it has learnt to disentangle the pathological and anatomical factors of a subject. In a further application to MS, Güllmar et al. [122] used a StyleGAN to learn disease progression in MS, particularly the neurodegenerative aspect, in a latent representation. This approach was trained on 71 consecutive axial slice positions of a T1w MRI and 41 consecutive axial slice positions of apparent diffusion coefficient (ADC) maps of 411 subjects from a proprietary dataset. To identify MS-specific features, a projection into a latent space was performed, resulting in independence between the disease factor and the variable age of a patient and the 2D slice position. Manipulations in latent space along the disease direction visualise both cortical atrophy and enlarged ventricles based on T1w sMRI, and, additionally, increasing lesion load in the periventricular region based on ADC maps with disease progression. Nonetheless, 2D sliced MRI might capture more information than derived volumetric data, but it does not necessarily capture local and global pathologies; extensions to 3D MRI inputs, although computationally expensive and requiring more data, are required. Bossa et al. [123] introduced a 3D-StyleGAN to learn a latent representation of PET images based on an additional encoder network. This latent representation was further compressed using principal component analysis (PCA) for dimensionality reduction to discover generating factors that can describe disease progression in Alzheimer’s disease. The brain amyloid evolution was then modelled on that low-dimensional data representation using non-parametric ordinal differential equations (ODEs) based on Gaussian Process (GP) regression. In an application to 1259 subjects with PET images from the ADNI database, the resulting model enabled the identification of different factors that encode amyloid load and the prediction of amyloid trajectories in individual patients.

**Fig. 5:**
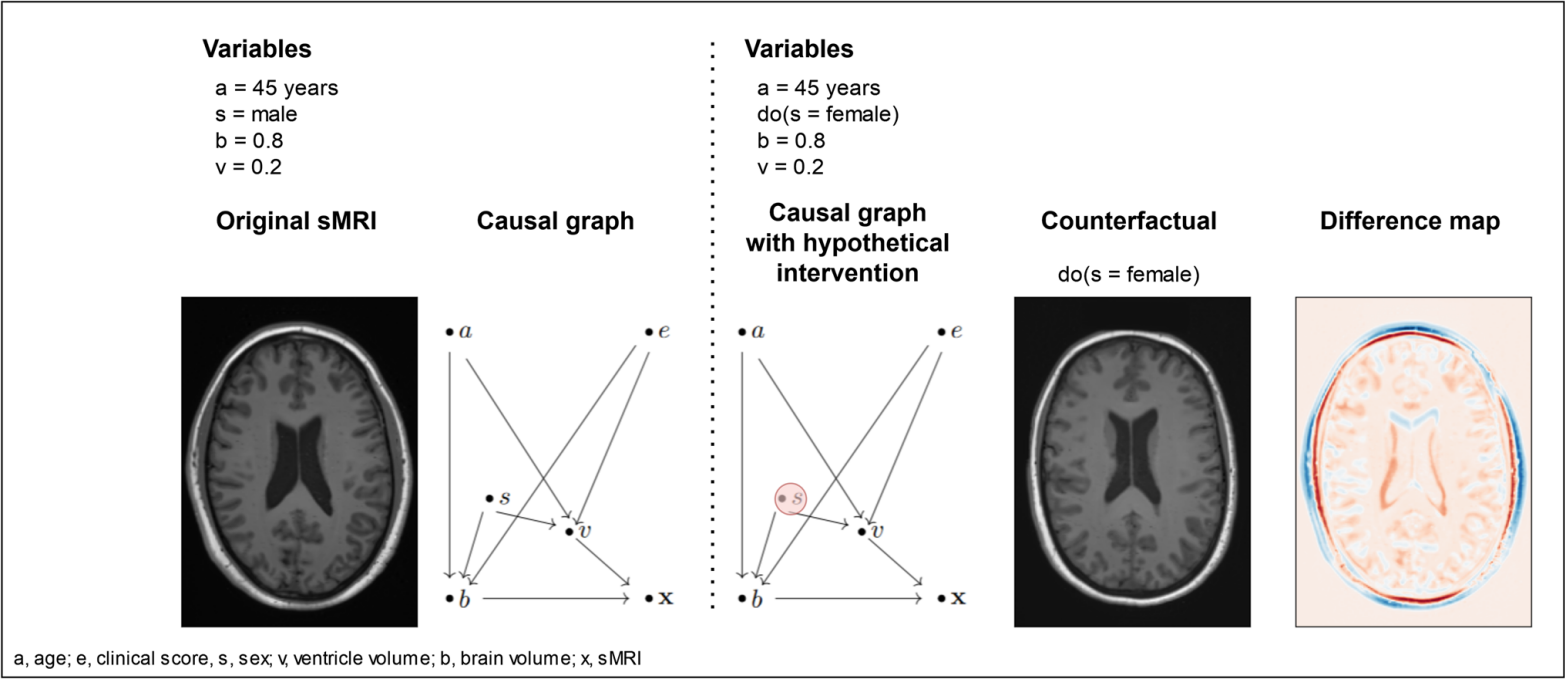
Original T1w MRI with corresponding variables and causal graph *(left)*, generated counterfactuals with hypothetical intervention on variable *sex* (*female* instead of *male*) and resulting difference maps between conterfactual and original T1w MRI *(right)*

A close connection to learning disentangled representations is the field of causal modelling. Palowski et al. [62] used the representation learning ability of VAEs to embed the generative model in a causal framework. The resulting deep structural causal model (DSCM), based on normalising flows and a VAE, allows for modelling hypothetical interventions and generating plausible counterfactuals by manipulating the latent representation using a predefined causal graph. In an application to the UK Biobank data [96], the causal graph included the variables sex, age, brain volume, ventricle volume, and the 3D T1w MRI. The core idea of generated counterfactuals is shown in Fig. 5.

These generated counterfactuals enable us to spatially measure the causal effect of factors such as ageing on brain structure using MRI. However, since the causal graph is predefined, the resulting causal effects rely on the correctness of the graph. While a causal discovery, i.e., methods for identification of the causal structure, can be used prior to the DSCM, the identifiability of an unambiguously causal structure based on observational data is not guaranteed [76]. Therefore, expert knowledge of causal interactions between the variables in the graph is required, particularly for medical applications. Potentially due to computational resources, the DSCM in this approach does not use the whole 3D T1w MRI but a 2D mid-axial slice [62], which is sub-optimal when measuring local or global causal disease effects on the brain structure. In addition, the VAE affects the quality of the generated data, resulting in blurry synthetic MRI scans. The latter was addressed in follow-up work by introducing a hierarchical variational autoencoder (HVAE) in the SCM framework to generate high-resolution synthetic data [124]. Additionally, a latent mediator model was introduced in the SCM, which enables a separate estimation of the direct, indirect, and total treatment effect [124]. Furthermore, Ribeiro et al. [124] included axial mid-slice T2 fluid-attenuated inversion recovery (FLAIR) MRI scans so that a synthesis between T1w and T2 FLAIR is possible for generation. Since both of these approaches were applied to UK Biobank, [107] extended the framework of [62] to study causal disease mechanisms of patients diagnosed with MS by generating counterfactuals of the brain structure in synthetic FLAIR MRI scans. The extensions to the causal graph included the disease-related variables Expanded Disability Status Scale (EDSS), symptom duration, lesion volume, and the use of multiple slices of MRI scans per subject to better spatially identify potential local or global causal effects [107]. While disease-specific effects, like lesion load, could partly be learned, the approach still suffers from its reliance on 2D slices instead of the whole 3D MRI scan to generate spatially plausible disease effects. A similar application to AD was proposed by abdulaal et al. [125].

Another approach to counterfactual reasoning was proposed by [126], which, in contrast to the DSCMs, relied on whole 3D MRI scans instead of 2D mid-axial slices. The introduced learn-explain-reinforce (LEAR) framework is based on a cGAN to generate the counterfactuals, a diagnostic model based on the same backbone, and a reinforcement learning-based model that uses the counterfactual maps for guidance to improve the performance of the discriminative diagnostic model. For training, 3D T1w MRI of 1538 subjects diagnosed cognitively unimpaired or either with stable mild cognitive impairment, progressive cognitive impairment, and AD from ADNI-1 and ADNI-2 were used. The resulting model learnt plausible counterfactual; however, only hypothetical intervention on the diagnosis was possible compared to more extensive causal graphs as in [62, 107, 125].

Overall, generative models can be helpful in the decomposition of neurological diseases. However, an evaluation of learnt representations and identified factors is difficult due to the lack of ground truth. Visual inspection, using manipulations of the latent space, or post-hoc correlation analyses are helpful but limited, especially for new anatomical or pathological insights. Towards the generation of a digital twin, the DSCM is a promising method to introduce a causal approach to the framework, which enables a causal understanding of disease effects. However, it currently seems limited to slice-based modelling due to computational reasons and requires a known causal graph without existing unknown confounders.

## Challenges

Besides the potential of generative modelling for neuroimaging applications in diseases of the central nervous system (“ Clinical applications of generative modelling” section), there are numerous challenges to further development and broad application of these approaches, e.g., in digital twins. In this section, we focus on the resources required for development (“Data and computational resources” section), model evaluation (“Evaluation” section), and the inherent risk to privacy (“Privacy” section).

### Data and computational resources

One of these challenges is the enormous amount of data and computational resources required for training and inference in these models. The relationships between generalisation performance, model complexity, data requirements and computational resources in deep neural networks, in general, are not well understood in theory [127, 128]. However, empirical scaling analyses in deep neural networks have been performed to understand these mechanisms [129–134] that can be described with a mathematical functional form called scaling laws, a particular form of power law. In particular, generative models seem to smoothly follow scaling laws with a predictable relationship between data set size, model complexity, and generalisation performance for a given computational budget.

Kaplan et al. [132] showed the power law relationships between the test loss, computational resources, dataset size and model size, which describe the reduction in generalisation performance based on an increase in the individual factors, given that they are not bottlenecked by the remaining two. In this example, a $10\times$ increase in the dataset size results in a $\sim 20\%$ decrease in the test loss, whereas a $10\times$ increase in model size results in a $\sim 16\%$ decrease in the generalisation performance within the considered limits. If the computational budget is limited, this type of power analysis can, for instance, be used to determine how this budget should be divided between an increase in the amount of data and the model size. The factors are increased in different proportions, where the proportions depend on different regimes of model and dataset sizes, but not in isolation, as this leads to diminishing returns. While better generative algorithms can improve the performances in a given domain, currently, the field focuses on scaling up models by increasing the dataset sizes in combination with the model complexity [135]. State-of-the-art generative models are already very complex, with billions of model parameters, and are trained on hundreds of millions or billions of data points, e.g., DALL-E2 uses 3.5 billion parameters and was trained on 650 million text-image pairs [16], GPT-3 has 175 billion parameters trained on 300 billion text tokens [11], Megatron-Turing NLG 530B has 530 billion parameters and a training set of 270 billion tokens [136]. This, in turn, comes at the cost of the computational resources for training and inference in these generative models, requiring specific hardware accelerators like graphical processing units (GPUs) or tensor processing units (TPUs). The training of the Megatron-Turing NLG 530B, for example, was performed on NVIDIA’s Selene supercomputer, hosting 4480 NVIDIA 80-GB A100 GPUs [136], which results in an estimated training time of $\sim$18 days [137]. There is often no transparency in this regard, as no information is provided on the resources used for training and inference in these models.

Due to recent advancements in the field, a so-called Mixture-of-Experts (MoE) layer was introduced to deep generative models (e.g., [138, 139]), which splits the computation into multiple expert sub-networks, leading to larger models and more efficient training given a fixed computational budget. In applications based on neuroimaging data, which are high-dimensional and, therefore, require a considerable amount of computation, the main bottleneck is data scarcity, in particular in databases containing patient data. This sounds paradoxical since one of the main arguments of generative models is data generation for small data regimes. Despite these innovations, the training and inference of these complex models require immense investment in computational infrastructure and electricity to operate, so the in-house use of these deep generative models entails considerable costs.

### Evaluation

An important challenge of paramount importance in generative modelling is the evaluation of these models, especially in high dimensions, which is essential to determine the effectiveness for its applications. In contrast to discriminative models used in classification, detection, segmentation, or regression, the downstream uses of generative models, due to their more general approach, are more difficult to characterise and define. The evaluation can be divided along the dimensions of utility or fidelity and privacy. In this section, we focus on the utility and fidelity dimension, while the privacy aspect is part of the “Privacy” section. The utility/fidelity evaluation of generative models is challenging and still an open problem because it requires the following dimensions: sample quality, sample diversity, and generalisation. Sample quality refers to the approximation ability of the generative model to the unknown data-generating distribution, sample diversity means the coverage of the data-generating distribution by the generative model, and generalisation means the generalisation ability of the model beyond the training data. Currently, no proposed metric captures all of these dimensions, but different focuses on different aspects in their evaluation.

A typical approach to evaluate the generative model is based on the likelihood of the observed data given the estimated model parameters. This type of evaluation is based on the close connection between the negative log-likelihood on the dataset and the Kullback–Leibler divergence, which is a popular distance measure between two probability distributions, here, between the generative model and the data-generating function. The negative log-likelihood on the test set is a proxy for fitting an estimate to the data-generating distribution, which is unknown. The problem with likelihood-based evaluation is that it is often computationally infeasible or, for implicit generative models (e.g., GANs), it is not even defined [2]. Further, Theis et al. [140] showed that likelihood is an implicit measure of diversity, but it does not necessarily correlate with sample quality.

Due to the challenges of comparing distributions in high-dimensional spaces, distances and divergences like the Inception score [141] or the Fréchet Inception distance (FID) [142] are often used heuristics for evaluation. While these scores only measure the closeness of the data and the model distribution, they are not suitable as diagnostic tools to identify potential failure modes. Here, improved precision and recall for distributions are used to measure the sample quality and sample diversity [143, 144]. Sajjadi et al. [143] define precision and recall using $P_r$, denoting the distribution of the observed data samples, and $P_g$, denoting the distribution of generated samples by the generative model. Precision is then defined as the probability that a random sample from the generated sample distribution $P_g$ falls within the space defined by $P_r$. Recall, on the other hand, is defined as the probability that a random sample of the real data distribution $P_r$ falls within the space defined by the distribution $P_g$ [143]. These scores or precision and recall are generally not assessed in isolation but are combined to evaluate the distributions produced by generative models.

Another approach to evaluate generative models is based on the evaluation of a classifier by comparing its performance on a downstream task using both the generated data set and the real-world data set (e.g., [145–147]). This form of evaluation involves various combinations of training and testing to measure the quality of the learned data-generating distribution, e.g., training a classifier on the real data samples and testing it on the generated synthetic data or the other way around to approximate distributional forms of precision and recall [148]. The main idea of this approach is to compare cross-dataset classification accuracy in downstream tasks as a proxy for the closeness of the real data distribution and the generated data distribution.

A human-based evaluation is another common approach in the evaluation of generative models. This process often involves visual inspection of the generated samples and a comparison to observed samples from the data distribution (e.g., [149]). This evaluation can be used in the optimisation process to align content with human preferences (reinforcement learning with human feedback, e.g., [150, 151]). While this type of evaluation is suitable for outputs for which it is difficult to define reliable metrics, it has the disadvantages of being time-intensive, lacking scalability and being prone to errors due to human raters.

While these approaches focus on evaluating the quality and diversity of examples, the generalisation ability of the generative model is not tested. To detect overfitting, a simple qualitative visual check is often performed by comparing semantically similar examples to detect possible memorisation. For likelihood-based methods, memorisation can be assessed by changes in the likelihood conditioned on the observation being included in the training data or not [152].

The evaluation along these dimensions is of imminent importance for safe and useful applications. However, due to the nature of the tackled problem in generative modelling, this remains a challenging open problem to be solved.

### Privacy

The evaluation of the privacy dimension is addressed in a separate section due to the sensitive nature of the data in medical applications. As discussed in the “Synthetic data generation” section, data synthesis can enable processes that would otherwise be impossible or difficult to realise, e.g., simplification of cooperation across legal borders [64]. However, while synthetic data in the medical image domain have often been simple test environments, deep generative models, offer new and complex possibilities in data synthesis [108]. Therefore, an evaluation of the privacy-utility trade-off may be required to consider whether deep generative models might, due to their capacity, pose a threat to privacy in applications in sensitive areas such as medicine. In this regard, questions such as the Ship of Theseus paradox [153] are currently being discussed in the generative modelling literature (e.g., [154]). The paradox questions whether the Ship of Theseus remains the same, even though all the original parts are replaced, over time, by new, identical parts, or whether it is an entirely different, new ship. A common misconception is that synthetic data is inherently private, and therefore, data privacy and compliance with legal frameworks like the General Data Protection Regulation (GPDR) or the Health Insurance Portability and Accountability Act (HIPAA) are negligible.

According to the GDPR, which regulates the protection of personal data, personal data is defined in Article 4 (1) GDPR as information relatable to an identified or identifiable person either directly or indirectly by a reference to an identifier [155]. In particular, processing medical imaging data raised ethical, legal and scientific challenges for years, e.g., in neuroimaging due to the uniqueness of structural or neural signatures allowing for re-identification of a person (e.g., [156–158]). Since an application of generative models to such medical imaging data, the question remains whether or not the generated data relates to the unique, original training data, allowing identification of the original patients, i.e., is the generated synthetic data pseudonymous or anonymous? None of the applications in the “Synthetic data generation” section mentions a privacy audit in this regard.

The GDPR defines pseudonymisation in Article 4 (5) GDPR as the processing of personal data, such that attribution to a data subject is not possible without additional information, which is held separately [155]. Anonymous data, in contrast, is defined in Recital 26 as information which is not relatable to an identified or identifiable person [155].

While generative models on natural images like DALL-E are often trained on hundreds of millions of images [16], the training data for medical image applications is significantly less (e.g., [108]). Deep neural networks in such data regimes are often over-parameterised and, therefore, prone to overfitting to the training data [159], i.e., the generative model could potentially memorise the training data and reproduce the training instances or incremental variations thereof. However, memorisation is not necessarily overfitting, as shown, e.g., by [152, 160], where memorisation of the training data occurs early in the dynamics of the training process before overfitting was observed due to influential observations or features in the training data. This memorisation behaviour in generative models poses a privacy risk due to information leakage and potential re-identification of patient information contained in the training set, from which only a pseudonymisation of the data can be derived, leading to an application of the GDPR. These attacks on generative models are known as membership attacks or membership inference [161, 162]. Targeted attacks or identifying matches have been studied extensively for different generative modelling approaches, which all have shown to be vulnerable to these attacks: e.g., VAEs [163], GANs [164, 165], diffusion models [166, 167]. In particular, diffusion models seem to pose a privacy risk because this model class is explicitly trained on memorisation and reconstruction of the training data, which raises the question of what generalisation means in these models or whether there is merely interpolation between memorised inputs, compared to other approaches like GANs [166]. Defences against such attacks, like deduplicating the training data [166] or training with differential privacy [168–171] can enforce privacy and mitigate privacy risks. This leads to the question of whether generative models can be used as an anonymisation tool. While the suitability of generative models for this purpose appears to vary, plain vanilla approaches offer no guarantee of privacy protection, although extensions can provide a remedy. Nevertheless, a risk audit along the privacy dimension is essential when applied to sensitive data; researchers and practitioners should be wary.

## Conclusion

Deep generative models offer enormous potential for neurological disease research, but the field is still in its infancy. In particular, their ability to synthesise data allows them to address current problems in the field, such as data scarcity, particularly pathological data. However, the statistical properties and the effects of synthesised data are not yet fully understood and require further research. In addition, the data is typically sensitive personal information that needs to be protected. Due to the capability of synthesising high-fidelity images and the potential risk of memorisation, an established privacy audit for generative models is required. Moreover, generative modelling enables a data-driven understanding of the underlying structures of diseases, but identification of the underlying structures is not guaranteed, and an evaluation is difficult given the nature of the problem definition.

However, the ability of generative models to learn a representation of the data within a causal framework, such as a deep structural causal model, makes them a key component in the concept of the digital twin, as it enables not only to measure causal effects but also to perform interventions or even counterfactuals, which are crucial for simulations within a digital twin. An efficient and scalable realisation of such a framework, with extended causal graphs within a patient, offers an enormous potential to transform medical decision-making towards precision medicine.

## Data Availability

No datasets were generated or analysed during the current study.
